# The Need and Safety of Mineral Supplementation in Adults with Obesity Post Bariatric Surgery—Sleeve Gastrectomy (SG)

**DOI:** 10.1007/s11695-021-05639-9

**Published:** 2021-08-04

**Authors:** Agata Wawrzyniak, Monika Krotki

**Affiliations:** Department of Human Nutrition, Institute of Human Nutrition Sciences, Warsaw University of Life Sciences (WULS-SGGW), 159C Nowoursynowska Str, 02-776 Warsaw, Poland

**Keywords:** Sleeve gastrectomy (SG), Adults, Mineral intake, Mineral supplementation

## Abstract

**Purpose:**

Most of the research indicated that daily dietary intake of minerals in SG patients was lower than the current recommendations. The aim of the study was to assess the need and safety of a mineral supplementation practice in adults with obesity, at 3, 6, and 9 months post bariatric surgery—sleeve gastrectomy (SG).

**Methods:**

The study included 24 women and 6 men. Based on a 4-day food record questionnaire, mineral and calorie intake was calculated at 3, 6, and 9 months after bariatric surgery (SG). Furthermore, an interview on supplement intake was also conducted.

**Results:**

It was found that in both men and women, there was a dietary intake deficiency of calcium (97% of respondents), potassium (97%), magnesium (83%), sodium (60%), and zinc (53%). In women, the deficiencies also included iron (50%) and copper (29%). Only 72% of the patients took dietary supplements. The applied supplementation did not adjust for the required intake of calcium in all of the patients, as well as the intake of magnesium in the male patients. Low intake of sodium and potassium were not supplemented and should be corrected by diet modification. The patients did not require supplementation of phosphorus or manganese, while male patients did not require iron or copper supplementation. The dietary and/or supplemental intake of minerals did not exceed the tolerable upper intake level (UL).

**Conclusion:**

The results of the study confirm the need to implement personalized mineral supplementation for bariatric surgery patients.

**Graphical abstract:**

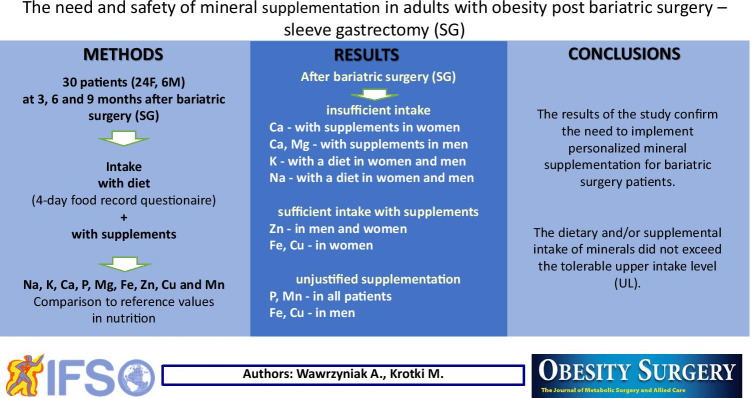

## Introduction

It is generally accepted that surgical treatment is a documented and effective method of treating pathological obesity, leading to long-term weight loss, as well as the remission of comorbidities (heart disease, metabolic disorders, lipid disorders, type 2 diabetes, sleep apnea, osteoarthritis) and decreasing risk of death [1–4]. In the world, the most common bariatric surgery is sleeve gastrectomy (SG) [2, 5]. This restrictive procedure [6] causes a limitation of food intake by decreasing the size of the stomach and reducing the ability to produce the hunger hormone—ghrelin—in the stomach. By decreasing the capacity of the stomach, the patient is unable to consume larger portions of food, as prior to the procedure, and the passage of food is accelerated through the digestive tract, which leads to a decrease in nutrient absorption and body mass [5, 7–9]. A significant side effect of all bariatric operations is nutritional deficiency, among other mineral insufficiencies, which can result in serious clinical consequences. It was observed that after SG and RYGB (Roux-en-Y gastric bypass), the mean daily dietary intake, primarily of calcium, magnesium, and iron, was lower than the current recommendations [10–14]. However, these studies have not shown whether the recommended mineral supplementation covers dietary deficiencies and whether or not there is a need to alter the supplementation practice due to further deficiencies or excessive intake. Thus, the aim of the study was the assessment of whether applied routine supplementation is sufficient in preventing mineral dietary deficiencies in patients having undergone bariatric surgery—sleeve gastrectomy in later post-surgical periods, i.e., at 3, 6, and 9 months after the procedure—and whether the scope of the administered supplementation exceeds safe levels.

## Methods

The study followed patients of the General, Oncological and Digestive Tract Surgery Department at the Medical Centre of Postgraduate Education at Orlowski Hospital in Warsaw, Poland. The study received the approval of the Bioethical Commission of the Centre of Postgraduate Education on April 12, 2017 (KB-W-382/2017), as well as the approval of the individual patients.

### Study Participants

The study concerned patients with obesity class III and greater, and with obesity class II with comorbidities, such as heart disease, metabolic disorders, lipid disorders, diabetes, sleep apnea, and osteoarthritis, prior to surgery. The patients undergoing the procedure met the following inclusion criteria: an age range between 18 and 60 years, BMI ≥ 40 kg/m^2^, or BMI 35–39.9 kg/m^2^ in persons additionally experiencing comorbidities. Exclusion criteria were as follows: inflammation of the digestive tract, chronic oesophagitis, gastric and duodenal ulcers constituting a risk of gastrointestinal bleeding, as well as digestive tract anomalies, severe heart disease and breathing difficulties, alcohol abuse, drug addiction, pregnancy, mental disorders, personality disorders, severe depression, a possible lack of patient engagement in the post-surgical treatment process, an inability to look after oneself, and a lack of due medical care from a caregiver post-surgery [6, 15, 16].

### Procedures

The study followed SG patients at 3, 6, and 9 months post-surgery. Participants completed a questionnaire about their age, dwelling place, education, job activity, and economic status. Dietary intake was assessed by a 4-day food record covering 3 working days and 1 non-working day. Patients reported all consumed products, meals, and drinks in grams/milliliters or in household measurements in three study periods. The portion size was defined using photographs of the products and meals showing three size options for each item [17]. The consumed foods were calculated into energy and nutrient units by Polish-developed computer software used to generate national food label composition tables [18]. In this paper, energy intake was reported for each stage of the study as separate mean values for women and men, while mineral intake (sodium, potassium, calcium, phosphorus, magnesium, iron, zinc, copper, and manganese) as a mean value of all three stages, for each subject or for women and men.

Throughout the postoperative follow-up period (including visits in months 3, 6, and 9 after surgery), all patients received dietary recommendations from a dietitian in the form of oral recommendations, a brochure or leaflet, and ready-made menus. The usage of dietary supplements was recommended to each patient by the operating surgeon, patients decided to choose and purchase one of the commercially available supplements. The amount of minerals taken in the form of supplements was calculated on the basis of a verbal interview at each stage of the study (product and dosage amount) and the product composition information on the food label. Usually patients took one tablet of MVMM product daily (Centrum, Vigor, Bodymax). The percentage of patients who did not meet the EAR (estimated average requirement) or AI (adequate intake) Polish recommendations [19] were determined during the 3-, 6-, and 9-month post-surgery time periods, taking into account only diet, as well as diet and supplements. Moreover, the tolerable upper intake level (UL) [20] was used to verify the safety of mineral intake.

### Statistical Analysis

Statistical analysis was carried out using the IBM SPSS Statistics 25 (SPSS Inc., Chicago, IL, USA) software package. Descriptive statistics and distribution normality testing of continuous variables were performed using the Shapiro–Wilk test; the results were presented as mean values, standard deviations, and percentages according to the type of variable. To assess the significance of difference between two independent groups, the Mann–Whitney U test (for continuous variables) or the chi^2^ test (for nominal variables) were used. To verify the significance between time periods or groups in repeatable measurements, a nonparametric Wilcoxon test (for 2 groups before and after the implementation of supplementation)/Friedman (for 3 period tests) was used, and in the case of the nominal variable for repetitive measurements, the McNemar test (for 2 groups)/W. Kendall (for 3 study periods) was used. The need for supplementation was verified using logistic regression analysis. The odds ratio (OR) and 95% confidence intervals (95% CI) were calculated. The reference category was the group without supplementation (OR = 1.00). The value of *α* = 0.05 was considered as statistically significant.

## Results

The study involved 30 (24 women and 6 men) bariatric SG surgery patients. The mean age of the female patients was 44 ± 10 years and for the male patients 50 ± 7 years (Table 1). There was no significant differences between the female and male patients regarding age, dwelling place, education level, and job activity of respondents.

**Table 1:** Participant characteristics

Characteristics	Women *(n* = *24)*	Men *(n* = *6)*	*p* value
Age (years)	44 ± 10*	50 ± 7	0.312**
Place of dwelling (% *n*)			
Village	13	0	0.655***
Town < 100,000	45	50	
City > 100,000	42	50	
Education (% *n*)			
Primary	21	50	0.218***
Secondary	54	50	
Higher	25	0	
Job activity (% *n*)			
No	33	17	0.400***
Yes	67	83	

^*^mean ± sd; **Mann–Whitney U test; ***chi^2^ test; *p* ≤ 0.05 statistically significant difference

Energy intake at 3, 6, and 9 months after surgery did not differ significantly among women (mean 1084 kcal/day) and among men (mean 1221 kcal/day), as well as the percentage of subjects taking dietary supplements (72% of respondents) (*p* > 0.05). This allowed the assessment of mineral intake between the group consuming only a food-based diet and the group consuming a diet with additional supplements, presented as the average of the three separate month periods (Table 2).

**Table 2:** Diet energy and usage of dietary supplements in post bariatric (SG) patients

Factor	Month after bariatric surgery			*p* value
	3rd *(n* = *30)*	6th *(n* = *30)*	9th *(n* = *30)*	
Energy intake (kcal)				
Women *(n* = *24)*	1083 ± 326*	1110 ± 293	1058 ± 287	0.185**
Men *(n* = *6)*	1419 ± 293	1123 ± 260	1121 ± 201	0.280
Multicomponent supplement used (%*n*)				
Women *(n* = *24)*	79	67	63	0.156***
Men *(n* = *6)*	83	83	67	0.368

^*^mean ± sd; **Friedman test; ***W. Kendall test; *p* ≤ 0.05 statistically significant difference

**Fig. 1:**
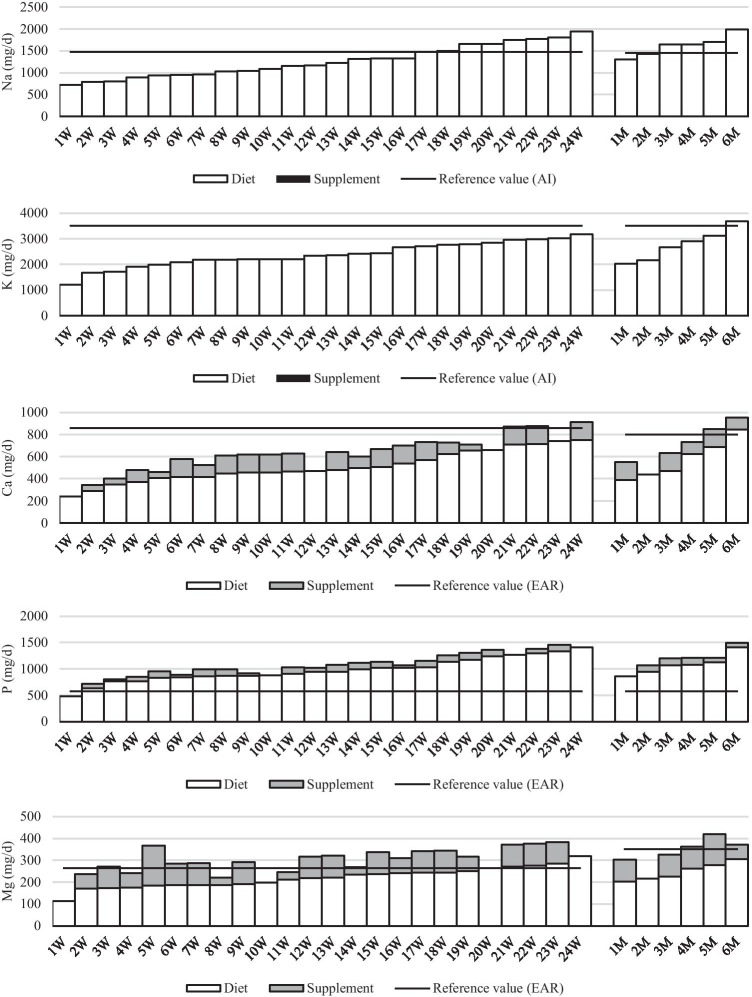
Mean dietary and supplement macromineral intakes by women **(W)** and men **(M)** tested at 3, 6, and 9 months after bariatric surgery (SG) (all data expressed as mg/day)

The limited energy intake after SG was associated with mineral deficiencies. Dietary sodium in 60% of subjects was lower than adequate intake. Dietary intake of potassium and calcium met recommendation only in the case of one male patient, while the recommendation for magnesium was met by only 5 women. In contrary, phosphorus intake from food in almost all of the patients (29) exceeded the nutritional recommendation (Fig. 1, Table 3).

**Table 3:** Mean mineral dietary intake without or with supplements in patients tested at 3, 6, and 9 months after bariatric surgery (SG)

Mineral intake	Women *(n* = *24)*		*p* value	Men *(n* = *6)*		*p* value	OR (95% CI)*** p value *n* = 30
	Without suppl	With suppl		Without suppl	With suppl		
Na							
Mean ± sd (mg/day)	1263 ± 361	1263 ± 361	1.00*	1621 ± 237	1621 ± 237	1.00*	-
Intake from food (%)		100			100		
Subject with intake < AI (%)	66.7	66.7	1.00**	33.3	33.3	1.00**	
K							
Mean ± sd (mg/day)	2377 ± 485	2377 ± 485	1.00	2761 ± 620	2761 ± 620	1.00	-
Intake from food (%)		100		100	100		
Subject with intake < AI (%)	100.0	100.0	1.00	83.3	83.3	1.00	
Ca							
Mean ± sd (mg/day)	510 ± 142	618 ± 164	< 0.001	576 ± 175	693 ± 191	0.038	5.80***
Intake from food (%)		72–100			71–100		(0.63–53.01)
Subject with intake < EAR (%)	100.0	87.5	0.083	83.3	66.7	0.317	0.119
P							
Mean ± sd (mg/day)	979 ± 227	1062 ± 238	< 0.001	1081 ± 191	1172 ± 209	0.038	1.00
Intake from food (%)		87–100			88–100		(0.06–16.76)
Subject with intake < EAR (%)	4.2	4.2	1.00	0.0	0.0	1.00	1.00
Mg							
Mean ± sd (mg/day)	220 ± 46	292 ± 64	< 0.001	247 ± 40	332 ± 70	0.039	15.17
Intake from food (%)		50–100			66–100		(4.09–56.25)
Subject with intake < EAR (%)	79.1	25.0	< 0.001	100.0	50.0	0.083	< 0.001
Fe							
Mean ± sd (mg/day)	7.7 ± 2.0	12.1 ± 3.6	< 0.001	7.8 ± 1.3	12.2 ± 3.5	0.039	7.00
Intake from food (%)		35–100			55–100		(1.38–35.48)
Subject with intake < EAR (%)	50.0	8.3	0.005	0.0	0.0	1.00	0.019
Zn							
Mean ± sd (mg/day)	7.1 ± 1.9	11.5 ± 3.8	< 0.001	7.7 ± 1.4	11.9 ± 3.3	0.039	10.29
Intake from food (%)		29–100			57–100		(2.56–41.37)
Subject with intake < EAR (%)	45.8	8.3	0.003	83.3	16.7	0.046	0.001
Cu							
Mean ± sd (mg/day)	0.8 ± 0.2	1.2 ± 0.3	< 0.001	1.0 ± 0.2	1.4 ± 0.3	0.039	8.83
Intake from food (%)		45–100			59–100		(1.01–76.96)
Subject with intake < EAR (%)	29.2	4.2	0.014	0.0	0.0	1.00	0.049
Mn							
Mean ± sd (mg/day)	3.4 ± 1.1	4.7 ± 1.5	< 0.001	4.4 ± 1.0	5.9 ± 1.5	0.034	1.00
Intake from food (%)		54–100			63–100		(0.06–16.76)
Subject with intake < AI (%)	4.2	4.2	1.00	0.0	0.0	1.00	1.00

^*^Wilcoxon test; **McNemar test; ***odds ratio (95% confidence interval) for subject intake > EAR/AI with vs. without supplementation; *p* ≤ 0.05 statistically significant difference; *EAR/AI* ref. value

Supplementation did not change the intake of sodium and potassium, as these minerals were not present in the supplements. The use of supplements did not affect the adequacy of phosphorus intake, because within the whole group undertaking the study, only one woman had intake that was too low. This woman did not take supplements throughout the period considered for this study. Although 25 patients (83%) were taking supplements with calcium (up to 162 mg Ca per day), the supplemental dosage was insufficient to achieve nutritional adequacy for calcium in 88% of the women and in 67% of the men. The supplemental contribution of calcium supply ranged from 0 to 30% and was too low. Among macrominerals, supplementation significantly increased the nutritional adequacy intake of only magnesium (OR = 15.17, *p* < 0.001), i.e. the total intake met the estimated average requirement in 53% of the patients. Nevertheless, total intake was too low in one third of the patients (in 25% of women and 50% of men).

**Fig. 2:**
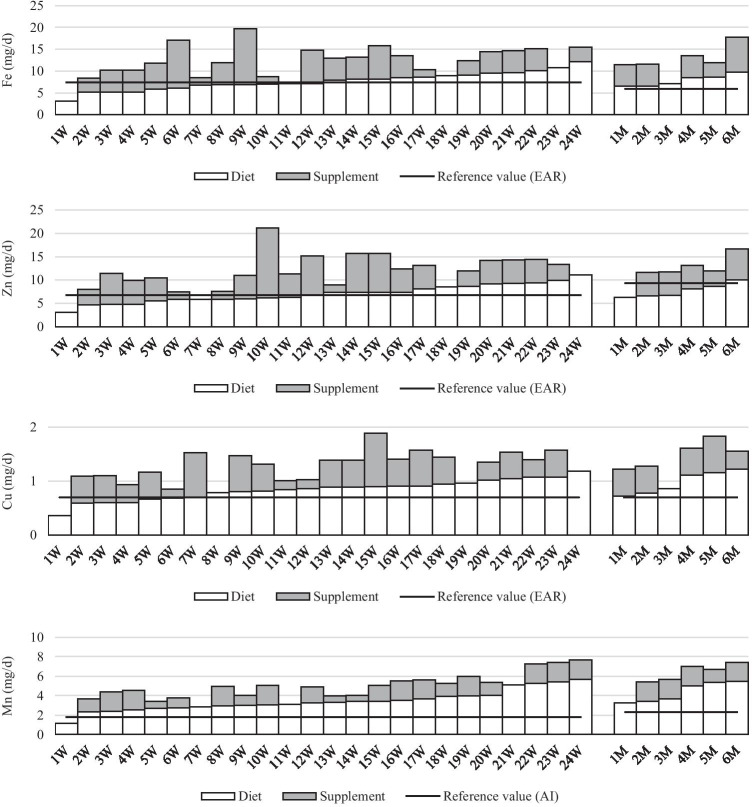
Mean dietary and supplement micromineral intakes by women **(W)** and men **(M)** tested at 3, 6, and 9 months after bariatric surgery (SG) (all data expressed as mg/day)

In the case of microminerals, the supply of iron and copper from a food-only diet caused deficiencies in 50% and 29% of the women, respectively. In men, the dietary intake of these minerals achieved adequacy and there was no need for supplementation. Deficient zinc intake from a food-only diet was observed in 53% of the respondents, i.e., 46% of the women and 83% of the men (Fig. 2). Supplementation significantly improved the intake of iron, zinc, and copper in women. Deficient intake of these elements was found in only 8% (iron, zinc) and 4% (copper) of the women, who did not use supplements. Similarly in men, deficient zinc intake was observed in only one man who did not use supplements. Manganese supplementation occurred to be unjustified for all respondents, except for 1 person, as manganese intake from a food-only diet met the recommendation without supplementation.

Only in the case of women did the intake of iron (in 17%), zinc (in 25%), and copper (in 8%) from supplements exceed the intake of these minerals from the food diet; however, none of these elements exceeded the safe UL.

## Discussion

The main observations of the study revealed that (1) the limited energy intake after sleeve gastrectomy is associated with irregularities of mineral intake, and patients require personalized supplementation; (2) the applied supplementation did not adjust to the required intake of minerals in patients; nevertheless, none of the patients exceeded the UL intake of minerals with diet and supplements.

### Energy and Supplement Intake in SG Patients

It seems that the most accessible and understandable form of nutritional recommendation for post bariatric surgery patients is the nutritional pyramid developed by Moizé et al. [21]. At the base of the pyramid is daily vitamin and mineral supplementation, because the result of a restrictive-type bariatric surgery is associated also with a deficiency in the intake of individual nutrients [22].

Similar or even lower values of energy intake, than the values presented in our study, were also observed by Gjessing et al. [22], Moizé et al. [10], Verger et al. [11], Jastrzębska-Mierzyńska et al. [13], and Hosseini-Esfahani, et al. [14]. Soares et al. [23] documented that patients did not follow the recommendations laid out in the nutritional pyramid. The subjects were not systematically taking vitamin and mineral supplements and consumed too little fruits and vegetables. Schiavo et al. [24] showed that after 3, 6, 9, and 12 months after SG, the percentage of people who did not follow the recommended diet was 39%, 45%, 51%, and 74%, respectively, and those who did not follow the recommended supplementation was 43%, 51%, 59%, and 67%, respectively. Nonadherence to the recommendations regarding diet and vitamin-mineral supplementation after bariatric surgery has also been proven by other authors [3, 4, 25–31].

### Mineral Intake and the Need for Supplementation in SG Patients

Patients’ documented calcium intake before and/or after SG surgery was deficient [10, 12–14]. Moizé et al. [10] showed insufficient calcium intake through the diet, which concerned 75–90% of the subjects before and within 60 months post SG. Deficiencies in calcium intake have been documented in the studies of Hosseini-Esfahani et al. [14], a year after SG surgery (in 97.5% of patients), and Jastrzębska-Mierzyńska et al. [13], in all patients 3 and 6 months post SG. In the study of Chou et al. [12], patients did not take calcium at the recommended amounts 5 years after SG surgery. Calcium deficiency in the diet and an insufficient supply of vitamin D is unfavorable for a patient, because it is associated with the risk of osteoporosis and bone fractures [32, 33]. That is why the American Society for Metabolic & Bariatric Surgery (ASMBS) recommends daily calcium supplementation for at least a year in the form of calcium citrate/carbonate in the amount of 1200–1500 mg post-surgery SG, RYGB (Roux en-Y gastric bypass), and AGB (adjustable gastric banding) [1, 34]; however, none of the patients implemented the supplementation at a level recommended by the ASMBS.

The incorrect intake of magnesium through diet, before and after surgery (after 6, 12, 24, 48, and 60 months), has been described in 87–100% of subjects in the Moizé et al. [10] study. Similarly, in the research of Jastrzębska-Mierzyńska et al. [13] and Hosseini-Esfahani et al. [14], deficiencies in magnesium intake were observed in 90% to 100% of respondents. Magnesium deficiencies may cause neuromuscular and cardiovascular disorders, insulin resistance, and impaired secretion of this hormone [19, 35]. In our own research, supplementation with this element also turned out to be insufficient, especially in women whose dietary magnesium intake was below 200 mg/day and the magnesium supplement intake below 100 mg in a daily dose. In men, a supplemental dose above 100 mg/day enabled the effective reduction of nutritional deficiencies, due to their approximately 30% higher recommended intake value.

Low sodium intake very rarely leads to its deficiency in the body [19]. After the SG surgery, the participants consumed smaller amounts of processed meat rich in sodium, and usually they did not salt their meals. They justified their behavior with the observation that a greater sodium intake would increase water retention in the body and as a consequence slow down weight loss. Sodium intake in our study was significantly lower than in the obese population qualified for bariatric surgery in the publication of Jastrzębska-Mierzyńska et al. [36], but similar to that reported by Chou et al. [12]. In the general population, salt intake greatly exceeds the recommended amounts, and therefore sodium is not intentionally incorporated into dietary supplements. The same applies to phosphorus, whose consumption with food usually exceeds recommendations, so there is no need for supplementation. Phosphorus in supplements occurs only as a salt component being the source of other elements. The supplements taken by the patients did not contain potassium. The study participants consumed insufficient quantities of vegetables, fresh and dried fruit, and cereals, products that are sources of potassium in the diet. Due to the fact that the kidneys have a large capacity to modify the supply of this element, the consequences of insufficient potassium intake are very rare [19]. Also Jastrzębska-Mierzyńska et al. [36] showed that before surgery the intake of potassium in 74% of women and 85% of men was too low.

Dietary iron deficiency post bariatric surgery has been documented by Moizé et al. [10]. Nonadherent dietary iron intake concerned 42 to 88% of the subjects at various time periods (before SG, 6, 12, 24, 48, and 60 months post-surgery). In the studies of Jastrzębska-Mierzyńska et al. [13], none of the women during the 3rd and 6th month post-surgery consumed iron according to the reference value, and none of the men in the study consumed iron 3 months post SG surgery. In the Hosseini-Esfahani et al. [14] study, only 30% of patients consumed adequate amounts of iron through diet. Dietary iron deficiency intake was also reported by Chou et al. [12]. Reduced daily intake and impaired iron absorption pose a threat of anemia. Symptoms of iron deficiency in patients include constant fatigue, impaired appetite, and pale skin. Limited consumption of iron-rich products (meat, whole grains, and selected vegetables) also promotes deficiencies. The use of a multicomponent supplement is often not effective and requires a one-component supplement. If oral supplementation is insufficient, iron can be taken intravenously or even a blood transfusion is necessary in critical cases [37, 38]. The iron supplemental dosage recommended by the American Association of Clinical Endocrinologists/Obesity Society/American Society for Metabolic & Bariatric Surgery (AACE/TOS/ASMBS) to prevent iron deficiency after SG is at least 18 mg and people at risk of anemia, a dosage of 45–60 mg [34, 39]. In our study, regardless of the supplemental dose taken (not less than 5 mg), all women (except for one woman who did not use supplements) achieved adequacy of iron intake. Dietary zinc intake lower than recommended by the AACE/TOS/ASMBS [39] was demonstrated by Chou et al. [12], Hosseini-Esfahani, et al. [14] (in almost 90% of respondents), and Jastrzębska-Mierzyńska et al. [13] (in all patients), because the consumption of zinc-rich products such as meat, liver, rennet cheese, whole-grain bread, buckwheat, and eggs decreases after bariatric surgery [25]. Zinc deficiencies in the diet are a risk factor for decreased immunity and hypothyroidism [19]. In our study, insufficient zinc intake was observed only in patients who did not implement supplementation of at least 5 mg/day.

All researchers agree that supplementation post SG is necessary because the diet does not meet the recommended intake of some minerals, most often calcium, magnesium, and iron [10, 34, 40]. Most researchers propose systematic daily supplementation according to ASMBS guidelines for 1 year after surgery; however, some nutritional deficiencies in the body occur even a few years later [39].

A strength of this study is that the analysis took into account the need for supplementation. Existing studies do not assess to what extent supplementation practice corrects insufficient dietary intake. None of the studies covered such a broad range of minerals and long time period (3–9 months) after the procedure, as in our study. Moreover, other studies did not assess whether supplementation practice led to exceeding safe levels. Our study is subject to a number of limitations. The study was conducted on a small group of men, which is related to the fact that the majority of patients undergoing surgical treatment for obesity are women. The second important limitation is that the study considered mineral intake only and did not apply nutritional status parameters of the patients (blood levels were not measured). Another limitation was finishing the study 9 months after surgery, due to the fact that fewer women and no men had nutrition follow-up after 9 months. However, the results of this study confirm that supplementation with selected minerals post SG surgery is necessary and safe and also indicates that bariatric patients need individually tailored dietary consultations to prevent nutritional deficiencies.

## Conclusions

To improve the implementation of dietary recommendations in women, special attention should be paid to correct calcium intake with dietary supplements and in men to correct calcium and magnesium intake. In all of our patients, zinc supplementation as well as the supplementation of iron and copper in women properly adjusted deficient intake from food products. Both men and women should be encouraged to modify their diet to improve potassium and sodium intake as both minerals are not usually recommended in the form of supplements. Phosphorus and manganese supplementation proved to be unjustified in all subjects, as well as iron and copper in men.
